# Physical activity, sedentary behavior, and cognitive function in children and adolescents: a scoping review of dose-response relationships, screen time interactions, and public health implications

**DOI:** 10.3389/fnbeh.2026.1848119

**Published:** 2026-05-29

**Authors:** Ismail Dergaa, Wissem Dhahbi, Mohamed Amine Dergaa, Mortadha Razzak, Halil İbrahim Ceylan, Valentina Stefanica, Raul Ioan Muntean, Noomen Guelmami

**Affiliations:** 1 Higher Institute of Sport and Physical Education of Ksar Said, University of Manouba, Manouba, Tunisia; 2 Research Unit of Physical Activity, Sport, and Health, UR18JS01, National Observatory on Sport, Tunis, Tunisia; 3 Higher Institute of Sport and Physical Education of Kef, University of Jendouba, El Kef, Tunisia; 4 Department of Physical Education and Sports Teaching, Faculty of Sport Sciences, Atatürk University, Erzurum, Türkiye; 5 Department of Physical Education and Sport, Faculty of Sciences, Physical Education and Informatics, National University of Science and Technology Politehnica Bucharest, Piteşti University Center, Piteşti, Romania; 6 Department of Physical Education and Sport, Faculty of Law and Social Sciences, University “1 Decembrie 1918” of Alba Iulia, Alba Iulia, Romania

**Keywords:** academic performance, BDNF, brain development, children, cognitive function, dose-response, executive function, neuroplasticity

## Abstract

**Background:**

Physical inactivity among children and adolescents has reached critical levels worldwide, with approximately 81% of school-aged youth failing to meet the World Health Organization recommendation of at least 60 min of moderate-to-vigorous physical activity per day. At the same time, screen-based sedentary behaviors have increased substantially, raising concerns about their combined impact on brain development, cognitive processes, and behavioral regulation. Although extensive research exists, evidence regarding dose–response relationships, neurobiological mechanisms, and the moderating role of screen-related behaviors remains fragmented.

**Objectives:**

This scoping review aimed to systematically map the evidence linking physical activity and sedentary behavior with cognitive outcomes in children and adolescents, examine dose–response patterns, synthesize underlying neurobiological mechanisms (including brain-derived neurotrophic factor and neuroplasticity), and evaluate the moderating influence of screen time and smartphone-related behaviors.

**Methods:**

The review followed PRISMA-ScR guidelines and the Joanna Briggs Institute methodology. Six electronic databases (PubMed/MEDLINE, Scopus, Web of Science, SPORTDiscus, PsycINFO, and CINAHL) were searched for studies published between January 2000 and March 2026. Eligibility criteria were defined using the Population–Concept–Context framework. Two independent reviewers conducted screening, achieving strong inter-rater reliability (κ = 0.84).

**Results:**

Of 2,843 records identified, 60 studies met inclusion criteria across four thematic domains: physical activity and cognitive outcomes, dose-response parameters, neurobiological mechanisms, and screen-based sedentary behavior including nomophobia. Chronic physical activity, particularly in school-based and clinical ADHD settings, was consistently associated with executive function improvements across included systematic reviews and trials. Screen time exceeding 4 h per day was associated with anxiety, depressive symptoms, and attentional deficits in observational data. Evidence on exercise-induced BDNF upregulation, contingent on structured exposure (≥3 sessions/week, ≥12 weeks), is restricted to five pediatric RCTs and warrants cautious interpretation.

**Conclusion:**

This scoping review maps consistent associations between physical activity and improved cognitive function in youth, mediated through neurobiological pathways that vary in strength across study designs and populations. Excessive screen exposure is associated with cognitive risk in observational data. Integrated public health frameworks addressing physical activity, screen time, and sleep represent a priority direction for future longitudinal research and policy development.

## 1 Introduction

Physical activity is one of the most potent environmental modulators of brain development across the pediatric lifespan. As early as 2003, meta-analytic evidence confirmed a positive relationship between physical activity and cognition in children (Sibley and Etnier, 2003), and the evidence base has expanded substantially since that foundational finding. Systematic reviews now document that chronic physical activity programs improve inhibitory control, working memory, cognitive flexibility, and academic performance in school-age populations (Donnelly et al., 2016; Alvarez-Bueno et al., 2017; Haverkamp et al., 2020). The neurobiological substrate of these effects involves exercise-induced upregulation of brain-derived neurotrophic factor (BDNF), hippocampal neurogenesis, prefrontal cortex remodeling, and enhanced functional connectivity within cognitive control networks (Hillman et al., 2008; Chaddock-Heyman et al., 2016; Rico-González et al., 2025). Aerobic fitness in children is directly associated with greater hippocampal cerebral blood flow (Chaddock-Heyman et al., 2016), a structural correlate of the memory and learning benefits documented in behavioral studies. These neurophysiological adaptations do not occur incidentally; they depend on adequate exposure, sustained over time, at intensities that activate the relevant biological cascades (Ben Ezzdine et al., 2025; Rico-González et al., 2025).

Against this evidence base, global trends in youth physical activity are deeply concerning. Data compiled from 298 population-based surveys involving 1.6 million adolescents confirm that 81.0% of school-going adolescents aged 11–17 years do not meet the minimum physical activity threshold of 60 min of moderate-to-vigorous intensity activity per day, with girls (84.7%) substantially less active than boys (77.6%) (Guthold et al., 2020). This deficit is not improving: between 2001 and 2016, the prevalence of insufficient physical activity remained stable in girls and declined only marginally in boys (Guthold et al., 2020). Physical inactivity is estimated to cost global public health systems approximately US$300 billion between 2020 and 2030 if current trends continue, encompassing direct healthcare costs and productivity losses attributable to activity-related chronic disease (Bull et al., 2020; van Sluijs et al., 2021). Among children and adolescents specifically, insufficient activity predicts adiposity, cardiometabolic risk, reduced cardiorespiratory fitness, and, critically, poorer cognitive and academic outcomes (Chaput et al., 2020; Wilhite et al., 2023; Kerr et al., 2025). The public health burden of cognitive underperformance in youth is not a secondary concern relative to physical health metrics; it is a primary driver of long-term educational, occupational, and economic disadvantage (Haverkamp et al., 2020; He et al., 2025).

The emergence of screen-based sedentary behaviors as a dominant feature of modern childhood and adolescence compounds this problem through a dual mechanism: displacing time available for physical activity, and exerting independent negative effects on cognitive development (Li et al., 2022; Muppalla et al., 2023). Systematic reviews confirm that screen-based sedentary time, particularly recreational screen use, is negatively associated with executive function in children and adolescents, with effects distinct from those of non-screen sedentary time (Li et al., 2022; Bustamante et al., 2023). At the population level, screen time exceeding 4 h daily is consistently associated with anxiety, depression, attention-deficit/hyperactivity disorder symptoms, and behavioral conduct problems in US children and adolescents (Dai and Ouyang, 2026). The neurobiological evidence further implicates smartphone and internet addiction in structural and functional alterations of the prefrontal cortex, insula, and anterior cingulate cortex in adolescents, with 21 fMRI studies collectively documenting impairments in cognitive control related to rewards processing and executive function (León Méndez et al., 2024). Nomophobia, the fear or discomfort associated with not having access to a smartphone, has transitioned from an emergent phenomenon to a prevalent condition affecting 15.2%–99.7% of young adults across published studies (Notara et al., 2021), with evidence that physical activity attenuates its severity (Aydin and Kuş, 2023; Mechraoui et al., 2023). These converging trends create what has been described as a bidirectional threat to pediatric cognitive health: physical activity is declining while screen-based displacement is rising, and each change reinforces the other.

Existing systematic reviews address components of this landscape in isolation. Reviews on physical activity and executive function (Alvarez-Bueno et al., 2017; Cabral et al., 2025; Mello et al., 2025), on sedentary behavior and cognition (Li et al., 2022; Bustamante et al., 2023), on screen time and development (Muppalla et al., 2023; Sticca et al., 2025), on BDNF and exercise in children (Rico-González et al., 2025), and on smartphone addiction and cognitive control (León Méndez et al., 2024) each contribute important evidence but do not collectively produce an integrated map of the evidence territory. Prior reviews have addressed these domains separately, without mapping the shared developmental context in which declining physical activity, expanding screen use, and inadequate sleep jointly shape cognitive outcomes in children and adolescents. No prior scoping review has mapped, within a single pediatric developmental framework, the evidence linking PA dose-response parameters, exercise-induced neurobiological mechanisms, and screen-based sedentary behaviors to cognitive function in children and adolescents aged 5–17 years. Where evidence from young adult or mixed-age studies is cited, its applicability to the pediatric age range is explicitly qualified. This evidence map is intended to support policy-makers, educators, clinicians, and public health professionals working at the intersection of youth physical activity and cognitive health. The present scoping review addresses this gap by pursuing four objectives: (i) characterize the volume, design, and geographic distribution of research on physical activity, sedentary behavior, and cognitive function in children and adolescents published between January 2000 and March 2026; (ii) map dose-response relationships and identify activity type, frequency, intensity, and duration parameters most consistently associated with cognitive benefit; (iii) synthesize the neurobiological mechanisms through which physical activity exerts cognitive effects, with particular attention to BDNF signaling and exercise-induced neuroplasticity; and (iv) characterize the cognitive consequences of sedentary behavior, screen time exposure, and smartphone-related behaviors, and identify the extent to which physical activity mitigates these risks.

## 2 Materials and methods

### 2.1 Study design

This scoping review was conducted in full accordance with the PRISMA extension for Scoping Reviews (PRISMA-ScR) (Tricco et al., 2018) and the updated Joanna Briggs Institute Reviewer’s Manual for scoping reviews (Peters et al., 2020). Scoping review methodology is appropriate for this research question because the objective is to map the breadth and heterogeneity of available evidence, identify conceptual and empirical gaps, and synthesize cross-domain findings rather than estimate a pooled effect size from a homogeneous study population. The review protocol was registered on the Open Science Framework prior to literature searching (OSF registration^1^).

### 2.2 Eligibility criteria: PCC framework

Eligibility was defined using the Population-Concept-Context (PCC) framework as recommended for scoping reviews (Peters et al., 2020). The full criteria are presented in Table 1.

**TABLE 1:** Eligibility criteria using the Population-Concept-Context (PCC) framework.

Component	Inclusion criteria	Exclusion criteria
Population	Children and adolescents aged 5–17 years; healthy populations; clinical populations with neurodevelopmental disorders (ADHD, ASD); overweight/obese youth; youth athletes	Adults aged 18 years and over; exclusively animal studies; studies with no data extractable for the target age range
Concept	Physical activity (structured exercise, school-based PA, unstructured play) and/or sedentary behavior (screen time, sitting time, nomophobia, smartphone addiction) as exposure; cognitive function (executive function, memory, attention, academic performance) as outcome; neurobiological mechanisms (BDNF, neuroplasticity, brain structure) as explanatory framework	Studies addressing exclusively adult or older adult populations; studies with no cognitive or brain outcome measure; purely physiological studies with no cognitive component
Context	School, home, community, recreational, and clinical settings in any country, any socioeconomic or geographic context	Occupational or workplace contexts exclusively
Publication	English-language peer-reviewed articles; systematic reviews, meta-analyses, narrative reviews, original research, scoping reviews; published January 2000 to March 2026	Conference abstracts without full text; non-peer-reviewed reports; editorials without empirical content

### 2.3 Information sources

Six electronic databases were searched: PubMed/MEDLINE, Scopus, Web of Science, SPORTDiscus, PsycINFO, and CINAHL. The search period spanned from January 2000 through March 2026. The reference lists of all included full-text articles were hand-searched to identify additional sources. The lower date boundary of January 2000 was selected to include the foundational meta-analytic literature that established the physical activity-cognition relationship in children (Sibley and Etnier, 2003), while capturing the full arc of subsequent evidence development through the screen time era.

### 2.4 Search strategy

The search combined three conceptual blocks using Boolean operators: physical activity and sedentary behaviors terminology; cognitive function and brain outcomes; and population specifiers. The PubMed/MEDLINE core string was: ((“physical activity” OR “exercise” OR “sport” OR “sedentary behav*” OR “screen time” OR “sitting time” OR “nomophobia” OR “smartphone addiction”) AND (“cognitive function” OR “executive function” OR “working memory” OR “inhibitory control” OR “cognitive flexibility” OR “academic performance” OR “attention” OR “brain” OR “BDNF” OR “neuroplasticity”) AND (“children” OR “adolescents” OR “youth” OR “pediatric” OR “pediatric” OR “school-age”)). Database-specific controlled vocabulary terms and MeSH headings were incorporated where available. The complete search strings for all six databases are presented in Table 2. The term “nomophobia” was applied only in PubMed/MEDLINE and Scopus strings, where preliminary scoping confirmed the highest indexed yield for this construct; in Web of Science, SPORTDiscus, PsycINFO, and CINAHL, the equivalent construct is predominantly indexed under “smartphone addiction” and “problematic smartphone use,” both of which were retained across all six database strings.

**TABLE 2:** Search strings were applied across six electronic databases.

Database	Search string
PubMed/MEDLINE	((“physical activity” OR “exercise” OR “sport” OR “sedentary behav*” OR “screen time” OR “nomophobia” OR “smartphone addiction”) AND (“cognitive function” OR “executive function” OR “working memory” OR “inhibitory control” OR “academic performance” OR “BDNF” OR “neuroplasticity”) AND (“children” OR “adolescents” OR “youth” OR “school-age”))
Scopus	TITLE-ABS-KEY((“physical activity” OR “exercise” OR “sedentary” OR “screen time” OR “nomophobia”) AND (“cognitive function” OR “executive function” OR “academic performance” OR “brain” OR “BDNF”) AND (“children” OR “adolescents” OR “youth”))
Web of Science	TS = ((“physical activity” OR “exercise” OR “sedentary behav*” OR “screen time”) AND (“cognitive function” OR “executive function” OR “working memory” OR “neuroplasticity”) AND (“children” OR “adolescents”))
SPORTDiscus	DE “physical activity” AND (“cognitive function” OR “executive function” OR “brain” OR “BDNF”) AND (children OR adolescents OR youth)
PsycINFO	(physical activity OR exercise OR sedentary behavior OR screen time) AND (cognitive function OR executive function OR working memory OR academic performance) AND (children OR adolescents)
CINAHL	MH “Exercise” OR MH “Sedentary Lifestyle” AND (“cognitive function” OR “executive function” OR “academic performance”) AND (children OR adolescents)

### 2.5 Selection process

**FIGURE 1:**
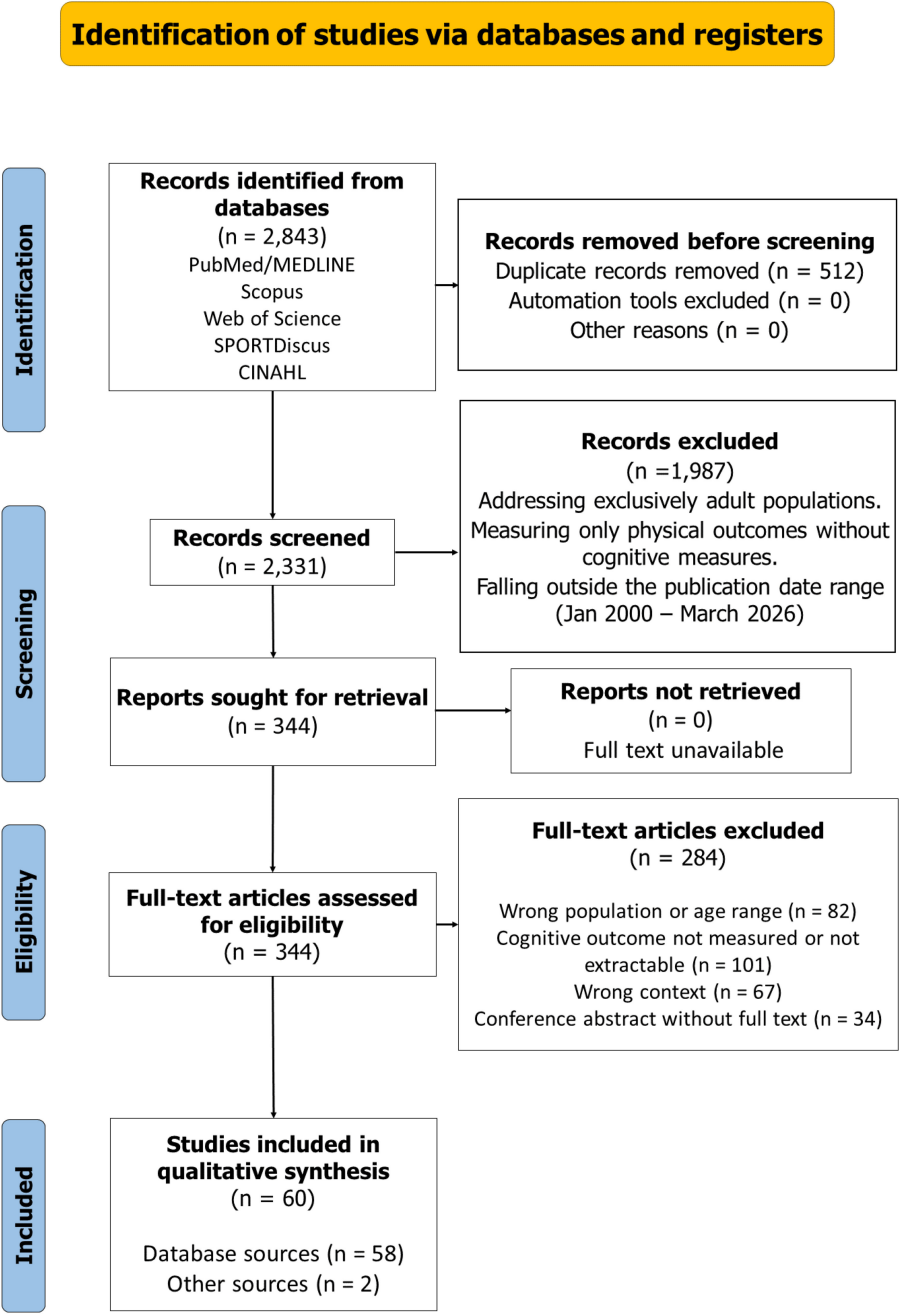
PRISMA-ScR flow diagram illustrating the identification, screening, and selection process for the scoping review. Six databases (PubMed/MEDLINE, Scopus, Web of Science, SPORTDiscus, PsycINFO, CINAHL) were searched for the period January 2000 to March 2026. Records identified: 2,843; duplicate records removed: 512; records screened (title/abstract): 2,331; records excluded at title/abstract: 1,987; full-text records assessed for eligibility: 344; full-text records excluded: 284 (wrong population or age range: *n* = 82; cognitive outcome not measured or not extractable: *n* = 101; wrong context: *n* = 67; conference abstract without full text: *n* = 34). Database sources (*n* = 58); sources identified through reference list hand-searching (*n* = 2); total 60 sources included.

Retrieved records were imported into Covidence systematic review software for duplicate removal and screening management. Two independent reviewers (I.D. and N.G.) screened all titles and abstracts against the PCC eligibility criteria. Disagreements were resolved by consensus; where consensus was not reached, a third reviewer (W.D.) adjudicated. Inter-rater reliability at the title and abstract stage was measured using Cohen’s kappa (kappa = 0.84, indicating strong agreement). Full-text assessment was conducted by the same two primary reviewers for all records passing initial screening. A standardized reason for exclusion was recorded for every excluded full text. The PRISMA-ScR flow diagram is presented in Figure 1.

### 2.6 Data charting process

Data were charted using a standardized extraction form developed iteratively and piloted on five randomly selected articles before full deployment. Extracted variables included: first author and year; country of corresponding author; study design; age range and sample characteristics; physical activity and/or sedentary behavior exposure; cognitive outcome measured; key quantitative findings; neurobiological mechanisms reported; limitations; and conclusions. Data charting was conducted independently by two reviewers (I.D. and M.A.D.) with discrepancies resolved through discussion. W.D. served as arbiter for unresolved discrepancies.

### 2.7 Data synthesis

Descriptive synthesis characterized the volume and distribution of included sources by publication year, country, study design, and thematic domain. Thematic synthesis organized findings across four domains identified during charting: physical activity and cognitive outcomes; dose-response relationships; neurobiological mechanisms; and sedentary behavior, screen time, and cognitive risk. Quantitative data were tabulated narratively where reported; meta-analysis was not conducted because the heterogeneity of included designs, populations, and outcome measures precludes pooling within this scoping review.

### 2.8 AI usage statement

Claude (Anthropic, version Sonnet 4.6) was used to assist with reference organization and structural drafting during manuscript preparation. The corresponding author (I.D.) conceptualized the review, led the literature search, performed data charting, wrote and critically revised the full manuscript, and assumes complete scientific and ethical responsibility for all content. All AI-assisted outputs were reviewed, edited, and verified by the author team before submission.

## 3 Results

### 3.1 Study selection

The six-database search retrieved 2,843 records. After removal of 512 duplicates, 2,331 unique records underwent title and abstract screening. Of these, 1,987 were excluded for addressing exclusively adult populations, measuring only physical outcomes without cognitive measures, or falling outside the publication date range. The remaining 344 full-text articles were assessed for eligibility. Of these, 284 were excluded: wrong population or age range (*n* = 82), cognitive outcome not measured or not extractable (*n* = 101), wrong context (*n* = 67), and conference abstract without full text (*n* = 34). Database sources (*n* = 58); sources identified through reference list hand-searching (*n* = 2); total 60 sources included. The PRISMA-ScR flow diagram is presented in Figure 1.

### 3.2 Characteristics of included sources

The 60 included sources span January 2003 to March 2026. Publication volume accelerated markedly from 2018 onward, with seven sources published before 2010, 14 between 2010 and 2018, 27 between 2019 and 2023, and 12 in 2024–2026. Corresponding author affiliations represented 24 countries, with the highest contribution from China (*n* = 11), the United States (*n* = 10), Spain (*n* = 7), Tunisia (*n* = 8), and Australia (*n* = 5). Study designs included systematic reviews and meta-analyses (*n* = 28), narrative and scoping reviews (*n* = 12), original experimental studies (including randomized controlled trials; *n* = 14), and observational studies (*n* = 6). The distribution of included sources across thematic domains is presented in Table 3. Quantitative findings throughout this section are reported using the metrics employed in the original studies (Hedges’ g for school-based and general meta-analyses; SMD for clinical population trials; prevalence percentages for observational data); cross-metric comparisons are not intended and should not be drawn, given the absence of a common standardized scale and the absence of pooled analysis within this review.

**TABLE 3:** Distribution of 60 included sources across thematic domains, principal cognitive outcomes, and key quantitative findings.

Domain	*n*	Primary outcomes	Key quantitative findings	Primary limitations
Physical activity and cognitive function (broad reviews)	12	Executive function, memory, attention, and academic performance	Positive associations consistent across designs; larger effects for aerobic exercise vs. non-aerobic (Sibley and Etnier, 2003; Donnelly et al., 2016)	Heterogeneous measurement tools; publication bias concerns across older reviews (Donnelly et al., 2016; Bidzan-Bluma and Lipowska, 2018)
Dose-response and exercise type	11	Cognitive flexibility, working memory, and inhibitory control	School PA: cognitive flexibility g = 0.244 (95% CI: 0.116–0.373); working memory g = 0.123 (95%CI: 0.028–0.219) (Mello et al., 2025); aerobic dose-response: MVPA ≥ 60 min/day optimal (Wang et al., 2025)	Dose-response curves not fully characterized; acute vs. chronic effects conflated in several reviews (Cabral et al., 2025; Dixon et al., 2025)
ADHD, NDDs, and clinical populations	7	Inhibitory control, working memory, and cognitive flexibility	Inhibitory control SMD = −0.50 (*p* < 0.00001); working memory SMD = −0.50 (*p* = 0.004); cognitive flexibility SMD = −0.45 (*p* = 0.01) (Song et al., 2023); network meta-analysis confirms combined aerobic + coordination > aerobic alone (Li et al., 2023)	Small sample sizes; short follow-up durations in most trials (Peters et al., 2020; Liu et al., 2025)
Neurobiological mechanisms (BDNF, neuroplasticity)	8	BDNF levels, hippocampal volume, and white matter integrity	2/5 RCTs showed significant BDNF increases; successful protocols: neuromotor/martial arts, ≥3 sessions/week, ≥12 weeks (Rico-González et al., 2025); hippocampal CBF positively associated with aerobic fitness in children (Chaddock-Heyman et al., 2016)	Very limited RCT evidence for BDNF in children; adult literature does not extrapolate directly to developing brains (Milbocker et al., 2024; Rico-González et al., 2025)
Sedentary behavior and screen time	10	Executive function, attention, language, and socio-emotional development	Screen time ≥ 4 h/day associated with anxiety, depression, ADHD, and behavioral problems (Dai and Ouyang, 2026); screen-based SB is more negatively associated with EF than non-screen SB (Li et al., 2022)	Cross-sectional designs dominate; causality unconfirmed; content quality not consistently measured (Li et al., 2022; Bustamante et al., 2023)
Nomophobia and smartphone addiction	7	Cognitive control, attention, executive function (fMRI)	21 fMRI studies: impairments in cognitive control in ACC, insula, DLPFC regions (León Méndez et al., 2024); higher PA intensity associated with lower smartphone addiction risk (Pirwani and Szabo, 2025)	Mainly young adult samples; limited longitudinal fMRI data in adolescents (León Méndez et al., 2024; Zhang et al., 2025)
Combined movement behaviors (PA + SB + sleep)	5	Cognitive function, executive function, and academic performance	High PA + low SB + adequate sleep = best cognitive outcomes; SB impacts stronger in adolescents than in younger children (Wilhite et al., 2023)	Heterogeneous definitions of “meeting guidelines”; cut-offs vary across national recommendations (Singh et al., 2012; Wilhite et al., 2023)

### 3.3 Physical activity and cognitive function: evidence across domains

#### 3.3.1 Executive function

Executive function is the cognitive domain most consistently associated with physical activity in children and adolescents. Among the 14 original experimental studies included in this review, several directly tested acute and chronic physical activity protocols in school-based samples; their design-level evidence is reported alongside the meta-analytic syntheses where extractable. A 2017 systematic review and meta-analysis of RCTs reported that physical activity interventions improved executive function overall, with the strongest evidence for inhibitory control and working memory (Alvarez-Bueno et al., 2017). The dose-response relationship between aerobic exercise and executive function in children was characterized in a 2025 meta-analysis of 19 RCTs (total *n* = 18,650 children), which found that aerobic exercise volume of 30–60 min per session at moderate-to-vigorous intensity, performed at least three times per week, produced the most consistent improvements across all three executive function domains (Wang et al., 2025). School-based physical activity programs produced a statistically significant improvement in cognitive flexibility (Hedges’ g = 0.244; 95% CI: 0.116–0.373; *p* < 0.001; I^2^ = 0%) and working memory (g = 0.123; 95% CI: 0.028–0.219; *p* = 0.012) across 18 studies included in one meta-analysis (Mello et al., 2025). The physical education literature further indicates that exercise type matters: cognitively engaging physical activity, such as martial arts, dance, and team sports that require tactical reasoning, produces greater executive function benefits than aerobic exercise without cognitive demands (Cabral et al., 2025). Acute bout effects are less clear; a 2025 systematic review and meta-analysis of 15 studies found no statistically significant impact of a single acute bout of physical activity on executive function accuracy (Cohen’s d = 0.02; 95% CI: −0.04 to 0.07) in preadolescent children (Dixon et al., 2025), a finding that contrasts with the robust chronic effects documented above. These null acute-bout findings, together with the inconsistent BDNF responses across pediatric RCTs (only 2 of 5 trials yielded significant increases; Rico-González et al., 2025), underscore that the cognitive benefits of physical activity are neither universal nor independent of exposure parameters.

#### 3.3.2 Physical activity in children with ADHD and neurodevelopmental disorders

Physical activity exerts its most clinically significant cognitive effects in children with attention-deficit/hyperactivity disorder. A 2023 meta-analysis of 24 studies (914 participants) found that physical activity interventions improved all three core executive function domains in children and adolescents with ADHD: inhibitory control (SMD = −0.50; 95% CI: −0.71 to −0.29; *p* < 0.00001), working memory (SMD = −0.50; 95% CI: −0.83 to −0.16; *p* = 0.004), and cognitive flexibility (SMD = −0.45; 95% CI: −0.81 to −0.09; *p* = 0.01) (Song et al., 2023). Subgroup analyses in the same study identified intervention intensity, motor skill type, number of sessions, and weekly exercise volume as significant moderators of executive function outcomes, providing actionable prescription parameters for clinical practice (Song et al., 2023). A 2023 network meta-analysis of RCTs in children and adolescents with neurodevelopmental disorders (ADHD, autism spectrum disorder) found that combined aerobic and coordination exercise programs outperformed aerobic exercise alone for executive function outcomes (Li et al., 2023). Physical activity interventions also showed positive behavioral effects in children with autism spectrum disorder, with structured programs improving motor, cognitive, and socio-emotional functioning (Mnif et al., 2024). This observational primary study, along with the original exercise trial in overweight adolescents (Li et al., 2025; Ghouili et al., 2026), constitutes direct experimental evidence within the corpus; their findings align with meta-analytic trends but reflect smaller samples and context-specific designs. Children with obesity represent a further high-priority population: a ML-based nutritional and physical fitness screening framework demonstrated that exercise-induced improvements in fitness metrics track with cognitive performance in overweight adolescents (Li et al., 2025).

#### 3.3.3 Academic performance

Academic performance is the functional cognitive outcome with the most direct policy relevance in school-based physical activity programs. A 2022 review and meta-analysis of longitudinal and intervention studies showed a positive association between physical activity across school-age years and academic performance, with the strongest effects observed for mathematics (Haverkamp et al., 2020). School-based physical activity consistently produces improvements that do not come at the cost of academic time, a concern that has historically limited physical education provision in many educational systems (Singh et al., 2019; He et al., 2025). A 2025 systematic review and meta-analysis across 34 studies found that school-based physical activity was associated with improved academic achievement in children and adolescents, with effects moderated by intervention duration and baseline fitness (Liu et al., 2025). Expert panel synthesis has identified evidence-based recommendations for maximizing academic benefits: physical activity should be incorporated throughout the school day rather than confined to dedicated physical education time, and interventions should be sustained for at least one academic semester to produce reliable academic effects (Tricco et al., 2018). These recommendations are consistent with the dose-response evidence from executive function research, which identifies frequency and cumulative volume rather than single-session duration as the primary drivers of cognitive benefit (Wang et al., 2025).

### 3.4 Neurobiological mechanisms

Brain-derived neurotrophic factor is the primary molecular mediator linking exercise to cognitive function in developing brains. A 2025 systematic review of RCTs in children aged 5–12 years identified five qualifying trials (*n* = 385 participants), of which two (40%) demonstrated significant increases in BDNF following exercise interventions (Rico-González et al., 2025). Successful interventions were characterized by neuromotor-oriented activities or martial arts programs (such as Taekwondo), training frequencies of at least three sessions per week, and durations of at least 12 weeks (Rico-González et al., 2025). Given that only two of five qualifying RCTs demonstrated significant BDNF increases, these findings should be interpreted as preliminary rather than conclusive (Rico-González et al., 2025). Interventions in overweight or obese children elicited less reliable BDNF responses than those in healthy-weight populations, suggesting that metabolic context modulates the neurobiological response to exercise (Rico-González et al., 2025). The TrkB signaling pathway, activated by BDNF binding, initiates downstream cascades supporting synaptic plasticity, neuronal survival, and hippocampal neurogenesis (Ben Ezzdine et al., 2025; Romero Garavito et al., 2025). In the hippocampus, these processes directly translate into improvements in episodic memory consolidation and spatial navigation, both of which are measured in the academic performance literature as components of learning (Chaddock-Heyman et al., 2016; Milbocker et al., 2024). Aerobic fitness in children is independently associated with greater hippocampal cerebral blood flow (Chaddock-Heyman et al., 2016), and this structural correlate of memory function is detectable using MRI in children as young as 8–9 years, confirming that exercise-induced neuroplasticity is active throughout childhood, not only during adolescence.

Physical exercise also promotes white matter development by myelinating prefrontal tracts, supporting the rapid maturation of executive function networks across middle childhood and early adolescence (Hillman et al., 2008; Milbocker et al., 2024). A comprehensive review covering neuroplasticity mechanisms from 2017 to 2023 showed that aerobic activity increases circulating IGF-1, VEGF, and BDNF, collectively supporting angiogenesis, neurogenesis, and synaptic remodeling (Milbocker et al., 2024). These mechanisms operate through dose-dependent pathways: BDNF release increases with exercise intensity up to approximately 60%–70% of maximum oxygen uptake, after which the incremental benefit plateaus (Romero Garavito et al., 2025). The translation of these molecular findings into practical exercise prescriptions for children requires acknowledging that the BDNF literature in pediatric populations remains small, with only five RCTs meeting quality standards (Rico-González et al., 2025). Extrapolation from adult and animal data should be approached cautiously, and the field requires dedicated pediatric RCTs that measure biomarkers alongside cognitive outcomes.

### 3.5 Sedentary behavior, screen time, and cognitive risk

Screen-based sedentary behavior exerts adverse effects on cognitive development that are distinct from, and compounding with, the opportunity cost of displaced physical activity time. A systematic review of 16 studies (12 cross-sectional and four longitudinal) found that screen-based sedentary behavior was negatively associated with executive function in five of nine studies measuring this construct, while accelerometer-measured total sedentary time showed more inconsistent associations (Li et al., 2022). This distinction is important: the cognitive harm of sedentary time appears concentrated in screen-based, passively consumed media use rather than in sedentary non-screen activities such as reading or structured quiet work (Li et al., 2022; Bustamante et al., 2023). Among children under 6 years, a meta-analysis of 15 studies (*n* = 6,922 participants) found no statistically significant overall association between screen time duration and executive function, but identified content quality and passive versus active screen use as critical moderating factors (Bustamante et al., 2023).

At higher exposure levels, the cognitive and mental health risks of screen time are more clearly defined. Daily screen time of 4 h or more was associated with anxiety, depression, behavioral conduct problems, and ADHD diagnosis in US children and adolescents after adjusting for confounders in observational analyses (Dai and Ouyang, 2026); the cross-sectional design of the primary evidence limits causal inference. Physical activity and sleep were identified as parallel mediators of this association, such that children who met physical activity guidelines exhibited substantially attenuated cognitive and mental health risks from equivalent screen time exposures (Dai and Ouyang, 2026). A review of combined movement behaviors (physical activity, sedentary behavior, sleep) showed that the most favorable cognitive outcomes were associated with the triple combination of high physical activity, low screen-based sedentary time, and adequate sleep duration; meeting any two but not all three conditions produced intermediate cognitive outcomes (Wilhite et al., 2023). Sedentary behavior exerted stronger negative effects on adolescents than on younger children, and screen-based sedentary time was more harmful than total sitting time across all age groups (Wilhite et al., 2023).

### 3.6 Nomophobia, smartphone addiction, and cognitive control

Nomophobia, defined as the fear or discomfort associated with the unavailability of one’s smartphone, represents a newer but rapidly growing cognitive risk in adolescent populations. Prevalence estimates across 40 studies meeting systematic review inclusion criteria ranged from 15.2% to 99.7%, with higher rates in samples collected during or after the COVID-19 pandemic (Notara et al., 2021); notably, this systematic review was conducted predominantly in young adult populations, and direct extrapolation of the age-gradient finding to children and adolescents is not supported by the available evidence. Internet and smartphone addiction are associated with alterations in brain regions central to cognitive control: a systematic review of 21 fMRI studies found that both addictions were associated with impairments in cognitive control related to rewards processing (anterior cingulate cortex, insula, amygdala) and executive function (dorsolateral prefrontal cortex, frontal and parietal lobes) (León Méndez et al., 2024). These neural signatures overlap substantially with the brain regions that benefit most from physical activity, specifically the prefrontal cortex and anterior cingulate cortex, suggesting a partially shared neurobiological substrate (Hillman et al., 2008; León Méndez et al., 2024).

Physical activity has been identified in the nomophobia literature as a potential protective factor, though this evidence is predominantly cross-sectional and based on young adult samples. Higher physical activity levels and intensity were independently associated with lower smartphone addiction risk in university students, and aerobic exercise produced measurable reductions in nomophobia severity in intervention studies (Mechraoui et al., 2023; Pirwani and Szabo, 2025). A 2023 study from Tunisia demonstrated that physical activity attenuated the severity of nomophobia in student populations, supporting the protective hypothesis in a non-Western context (Mechraoui et al., 2023). The COVID-19 pandemic created a natural experiment documenting these relationships at scale: school closures and home confinement simultaneously reduced physical activity, increased screen time, and elevated nomophobia and smartphone addiction prevalence in adolescents worldwide (Aydin and Kuş, 2023; Taheri et al., 2023). Recovery of physical activity levels following lockdowns was associated with partial recovery of cognitive performance and a reduction in nomophobia symptoms in a longitudinal study (Taheri et al., 2023; Tao et al., 2023).

### 3.7 Sleep, physical activity, and cognitive performance

Sleep constitutes a third movement behavior domain that interacts with physical activity and screen time to determine cognitive outcomes. Adequate sleep duration is associated with better executive function, memory consolidation, attention regulation, and academic performance in children and adolescents, while sleep restriction impairs all these domains (Matricciani et al., 2019). Evening blue light exposure from screen devices advances melatonin suppression, delays sleep onset, and reduces total sleep duration in adolescents, creating a mechanistic pathway from evening screen use to next-day cognitive deficits (Dridi et al., 2025; Souissi et al., 2025). This pathway was confirmed experimentally: evening smartphone exposure in elite athletes impaired sleep quality and reduced next-day motor and cognitive performance compared to no-device conditions (Dridi et al., 2025; Souissi et al., 2025). Sleep restriction in elite adolescent athletes produced significant reductions in both cognitive and physical performance, with crossover designs confirming a direct causal relationship (Dridi et al., 2025).

From a public health standpoint, the combination of insufficient physical activity, excessive screen-based sedentary time, and poor sleep quality represents a convergent threat to cognitive development that single-domain interventions are poorly positioned to address (Matricciani et al., 2019; Wilhite et al., 2023). Meta-review evidence confirms that children’s sleep and health are reciprocally linked: physical activity promotes deeper, longer sleep, while poor sleep attenuates the cognitive recovery that underpins next-day learning capacity (Matricciani et al., 2019). Intervention programs that target all three movement behaviors simultaneously, building physical activity volume while reducing screen time and improving sleep hygiene, produce the most durable cognitive benefits in school-age populations (Wilhite et al., 2023).

## 4 Discussion

This scoping review provides a comprehensive evidence map linking physical activity, sedentary behavior, and cognitive function across childhood and adolescence. Sixty sources covering more than two decades of research indicate that the relationship between physical activity, sedentary behavior, and cognitive function in youth is bidirectional, dose-associated, and potentially modifiable. The available evidence, though consistent in direction, varies in strength across study designs; associations in favor of physical activity are robust in meta-analytic reviews, whereas the neurobiological evidence in pediatric populations, particularly regarding BDNF upregulation, remains preliminary. Three overarching conclusions emerge with practical consequences.

### 4.1 Physical activity is a dose-dependent cognitive intervention

The evidence is consistent that physical activity produces cognitive benefits through chronic, cumulative mechanisms rather than acute single-session effects. Chronic school-based programs produced significant gains in cognitive flexibility and working memory (Mello et al., 2025), while a single acute session produced no statistically meaningful effect on executive function accuracy (Dixon et al., 2025). This dissociation has direct implications for how physical activity is positioned in educational policy. Physical education periods concentrated on a few days per week, with long gaps in between, are less likely to produce cognitive benefits than distributed physical activity breaks integrated throughout the school day, a model supported by expert panel recommendations (Tricco et al., 2018). Additionally, not all chronic programs produced effects of equal magnitude; intervention heterogeneity in duration, intensity, and cognitive engagement format accounts for substantial variability in reported outcomes.

The type of physical activity matters as much as its volume. Cognitively engaging activities, particularly those that demand tactical reasoning, motor learning, and social coordination, produce greater executive function benefits than purely aerobic exercise (Cabral et al., 2025). Martial arts programs, team ball sports, and dance meet this criterion more fully than treadmill-based aerobic exercise, and these modalities were associated with BDNF responses in two RCTs from a pool of five qualifying trials that demonstrated significant increases (Rico-González et al., 2025). For children with ADHD, the meta-analytic evidence is more consistent than in general pediatric populations (Song et al., 2023), supporting physical activity as a promising non-pharmacological adjunct to established ADHD management pathways, though further RCTs with standardized outcome measures are needed. Dose parameters are now sufficiently defined to support clinical prescription: at least three sessions per week, at moderate-to-vigorous intensity, with each session lasting 30–60 min, over a minimum of 12 weeks, with cognitive engagement embedded in the activity format (Song et al., 2023; Rico-González et al., 2025; Wang et al., 2025).

### 4.2 Screen time constitutes a quantifiable cognitive risk

The threshold of 4 h of daily screen time represents the most consistent dose at which cognitive and mental health risks become statistically significant in population-based data (Dai and Ouyang, 2026). Below this threshold, the evidence is more heterogeneous, with content quality and passive versus interactive use emerging as stronger determinants of cognitive outcome than raw duration (Bustamante et al., 2023; Sticca et al., 2025). This nuance is important for public health messaging: a prohibition-focused approach to all screen time is inconsistent with the evidence and risks losing credibility with families. Evidence-based guidance should instead distinguish between passive, non-educational screen consumption and interactive, educationally purposeful screen use, while maintaining clear dose limits for the former.

The nomophobia and smartphone addiction literature adds a neurobiological dimension to these population-level findings. The consistent pattern of prefrontal and anterior cingulate cortex dysregulation documented across 21 fMRI studies (León Méndez et al., 2024) suggests that chronic high-intensity smartphone use in adolescents is not merely a time displacement problem but a potential modifier of brain development in regions that are still actively maturing during the second decade of life. Preliminary evidence suggests that physical activity may attenuate the behavioral severity of nomophobia (Mechraoui et al., 2023; Pirwani and Szabo, 2025); whether this reflects a reversal of underlying neural signatures in adolescents requires dedicated longitudinal neuroimaging investigation.

### 4.3 Combined movement behavior frameworks are essential

The clearest cognitive benefit accrues when high physical activity, low screen-based sedentary time, and adequate sleep are achieved together (Wilhite et al., 2023). The magnitude of cognitive benefit from meeting all three guidelines substantially exceeds that from meeting any two, and the harm from failing to meet any one is amplified when the other two are also unmet. This interactive structure means that single-domain interventions, whether targeting only physical activity or only screen time, will systematically underperform relative to integrated approaches that address all three simultaneously. School-based programs that offer structured physical activity, screen use governance during school hours, and sleep education for students and families are therefore more likely to produce durable cognitive gains than any single-behavior approach.

The bidirectional relationships between these domains are also clinically relevant. Evening screen use disrupts sleep through blue light mechanisms (Dridi et al., 2025; Souissi et al., 2025), inadequate sleep reduces next-day physical activity participation (Dridi et al., 2025), and low physical activity increases the risk of habitual screen engagement as a sedentary default (Musa et al., 2022; Aydin and Kuş, 2023). Interventions that break into this cycle at any single point face these reinforcing feedback loops. Breaking multiple links simultaneously, the design logic of integrated movement behavior programs is more likely to produce sustained behavior change. The public health cost of failing to achieve this is substantial: an estimated US$300 billion in health system costs attributable to physical inactivity between 2020 and 2030 (Bull et al., 2020), not including the long-term educational and productivity costs of impaired cognitive development during sensitive periods of brain maturation.

### 4.4 Future research priorities

Five research gaps emerge as the highest priority from this review. First, pediatric RCTs measuring BDNF alongside cognitive outcomes are urgently needed to close the translational gap between adult neurobiological evidence and practical exercise prescription for children (Rico-González et al., 2025). Second, longitudinal studies tracking combined movement behavior patterns from early childhood through late adolescence are required to identify sensitive periods for exercise-induced neuroplasticity and screen time harm (Milbocker et al., 2024; Sticca et al., 2025). Third, the neuroimaging literature on nomophobia and smartphone addiction has been conducted predominantly in young adults; dedicated fMRI studies in younger adolescents are required to establish whether the documented prefrontal and anterior cingulate alterations (León Méndez et al., 2024) are present in the age group at greatest developmental risk. Fourth, the dose-response evidence for cognitive outcomes remains incomplete at low activity doses; practically relevant research should characterize the minimum effective dose for populations unable to achieve the 60 min/day guideline, to inform step-wise public health targets (Chaput et al., 2020). Fifth, a consensus reporting standard for physical activity and cognition trials in children, analogous to CONSORT, would substantially improve cross-study comparability and the translation of evidence into guideline recommendations (Liu et al., 2025; Peters et al., 2020).

### 4.5 Limitations

Several methodological limitations qualify the conclusions of this review. The restriction to English-language publications introduces a language bias that may exclude relevant evidence from research communities publishing in other languages. The lower boundary of the January 2000 search captures the modern quantitative literature but excludes earlier observational and descriptive work. Heterogeneity in cognitive outcome measurement across studies, with no single standardized battery consistently applied, limits direct comparisons of effect sizes across domains. The scoping methodology intentionally does not weight evidence by quality, meaning that observational associations and RCT findings are mapped together without formal quality grading. The specific sub-domain of nomophobia and smartphone addiction in adolescents is a rapidly developing field, and some included studies reflect a young evidence base with predominantly cross-sectional designs and young adult rather than specifically adolescent samples. These limitations should be addressed by targeted systematic reviews with a narrower scope and rigorous quality assessment, building on the evidence map this review provides.

## 5 Conclusion

This scoping review maps consistent associations between physical activity and improved cognitive function in children and adolescents, with neurobiological pathways, including BDNF signaling, hippocampal neuroplasticity, and prefrontal white matter development, proposed as mediating mechanisms. The associations are dose-associated, with chronic engagement at moderate-to-vigorous intensity and embedded neuromotor complexity producing the most consistent benefits across included studies. Secondary meta-analyses within the corpus reported improvements in cognitive flexibility (Hedges’ g = 0.244) and working memory (g = 0.123) in school-based programs, and standardized mean differences of −0.45 to −0.50 across executive function domains in children with ADHD; these figures are extracted from specific included studies and do not represent pooled estimates generated by this review. No formal quality assessment of primary studies was conducted, and the strength of these associations should be interpreted within that methodological constraint.

Screen-based sedentary behavior is associated with cognitive and mental health risk in observational data, with daily exposures exceeding 4 h linked to anxiety, depression, and attention deficits. Neuroimaging evidence links smartphone addiction to alterations in the prefrontal and anterior cingulate cortices, predominantly in young adult samples; whether physical activity modifies these neural patterns in adolescents remains to be investigated. The most protective configuration for cognitive development combines high physical activity, low screen-based sedentary time, and adequate sleep; achieving all three simultaneously produces cognitive outcomes substantially superior to achieving any one or two in isolation. Public health policies that treat physical activity, screen time, and sleep as independent targets are misaligned with the interactive evidence structure this review has mapped. Integrated movement behavior frameworks, embedded in school curricula, family guidance, and healthcare recommendations, represent the evidence-based response to the compound threat of youth physical inactivity and screen time expansion. The global burden associated with 81.0% of adolescents failing to meet minimum physical activity recommendations underscores the urgency of integrated policy action; the evidence mapped in this review identifies physical activity promotion, screen time governance, and sleep support as three interconnected targets, the simultaneous pursuit of which is more likely to produce cognitive benefit than any single-domain approach, though this hypothesis requires validation through well-designed longitudinal trials.

## Footnotes

1
https://osf.io/hmsya/
